# Community-Led Assessment of Risk from Exposure to Mercury by Native Amerindian Wayana in Southeast Suriname

**DOI:** 10.1155/2012/674596

**Published:** 2011-11-15

**Authors:** Daniel Peplow, Sarah Augustine

**Affiliations:** ^1^Suriname Indigenous Health Fund, International Humanities Center, White Swan, WA 98952, USA; ^2^College of the Environment, University of Washington, Seattle, WA 98195, USA

## Abstract

This study was a collaboration between Western public health researchers and Suriname indigenous communities. The question asked was “how can Western researchers effectively engage traditional indigenous communities in Suriname, South America, in public health research”. The approach used a combination of Participatory Action Research methods in which “Western” researchers became participating observers in an indigenous-led research initiative. The Wayana communities of Puleowime (Apetina) and Kawemhakan (Anapayke) defined a single objective: determine for themselves whether they are at risk from exposure to mercury (Hg) contamination. Community members collected hair samples for analysis. Hair samples were analyzed using a portable Hg analyzer. Individual, community and hazard quotient indices were used to quantify risk. Results showed the Wayana were at a high lifetime risk of adverse effects from exposure to Hg. This study showed that the community-led approach is an effective way Westerners can engage indigenous communities and address serious public health threats. While factors that appealed to indigenous communities were identified, obstacles inherent to Western research methodology were also encountered.

## 1. Introduction

This study began in 2007 with one overarching objective, which was for Wayana communities in southeast Suriname to determine for themselves the risk of exposure to contaminants from mining, especially mercury (Hg), on their health. To this end, a community-led research design was created in partnership between community leaders in two Wayana villages and two non-governmental organizations. Community leaders represented the perspective and interests of community members and as such defined the context, the research problem and led the exploration of culturally appropriate public health solutions. Community leaders invited two non-governmental organizations to assist them in designing and carrying out an environmental risk assessment study. The Suriname organization Stichting Wadeken Wasjibon Maria (SWWM) provided guides, educators, and translators, whereas the USA-based Suriname Indigenous Health Fund (SIHF) provided expertise by a toxicologist, a sociologist, and a public health physician. 

Indigenous villages in Suriname's interior region are dependent on their traditional lands for hunting, fishing, farming, medicines, shelter, and their daily necessities such as clean water [1]. Many of these people have been displaced from their lands due to mining concessions [2]. Kawenhakan is an example. Families are relocating from the Suriname side of the Lawa River to the French Guiana side because of pollution of their traditional water source by gold mining activities. Gold mining activities release mercury (Hg) into the water. Methylation of inorganic Hg released by mining leads to fish contamination, and fish are the primary source of protein for these communities. Hg toxicity causes irreversible damage to the environment and to the health of the general population living in the region where mining occurs.

Since the end of the last century, gold mining in Suriname has been carried out at numerous sites and still continues to be practiced by international mining companies and smaller groups on terrestrial sites or by dredging operations directly in the rivers. In Suriname it is estimated that 20,000 kg/year Hg is discarded into the environment by small-scale and artisanal gold mining [3, 4]. Other studies estimate that gold mining releases 9,000–54,000 kgs of Hg in Suriname interior rainforest [5].This amount is orders of magnitude larger than other sources including Hg from bauxite refining and biomass [6]. Emissions by bauxite refining were estimated to be 500–600 kg/year in 2002-2003 and below 150 kg/year in 2005 from the “Suriname Aluminum Company.” Emissions from biomass burning are estimated to be about 30 kg/year. 

A range of health outcomes is expected when communities like the Wayana live close to resources valuable to mainstream society. Resource exploitation often affects indigenous people negatively either through exposure to environmental contamination or by restricting their access to forest areas that provide living space, medicinal organisms, food, building materials, and water [7]. The maintenance of traditional culture is thought to be a protective factor, especially for problems related to nutrition. 

**Figure 1:**
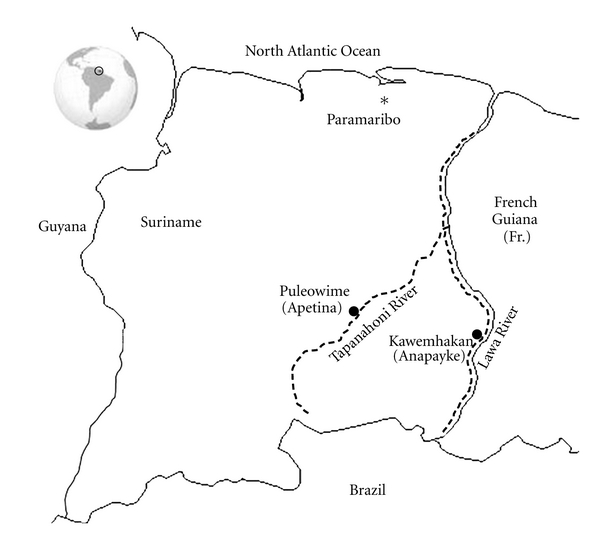
Map of Suriname showing location of communities that led the community-directed mercury risk assessment study.

Gold mining activities are mainly concentrated within an area of Eastern and Southeastern Suriname (Figure 1). This region is rich in minerals, including gold. And at the same time, it is rich in biodiversity and is inhabited by a variety of indigenous (Amerindian) and tribal (Maroon) communities. The significance of this study, conducted by the communities Puleowime (Apetina) and Kawemhakan (Anapayke), is not limited to the Greenstone Belt Region (GBR) because the direct impacts of mining go well beyond the geological boundaries of the GBR [8]. The impacts of gold mining in Suriname fall into four general categories: physical, chemical, biological, and social. Specific examples of impacts include the degradation of landscapes and soils, altered hydrogeological regimes, modification of surface drainage, degradation of drinking water resources, degradation of surface water by increased turbidity, contamination of land and water by solid and liquid waste and mercury, loss of natural habitats and biodiversity, loss of rare and endangered species, degradation of fisheries, and finally the degradation of the social integrity and physical health of indigenous communities living in the region where gold mining occurs. The villages of Puleowime (Apetina) and Kawemhakan (Anapayke) are home to the Wayana Tribe, a collective name for several ethnic groups including the Upului, Opagwana, and Kukuiyana [9, 10]. 

Mercury contamination is of particular importance to indigenous communities in the region. In neighboring French Guiana, a mercury risk assessment study was conducted in 1994, where hair samples were analyzed for Hg [11]. Results showed high levels of Hg, only in the native Amerindian communities living in the upper reaches of the Maroni River. This contamination probably reflects past and current gold mining activities in this area and is linked to the diet of these populations, of which fish is a main component. This population's health is a major concern for France because the Wayana is an ethnic group that may be vulnerable due to their particular way of life. 

In Suriname, numerous risk assessment studies reporting the effects of Hg pollution on public health have been performed. However, few studies have been published. Recent studies have shown high levels of Hg in people from interior communities on the Saramacca River [12]. Hg vapor from gold shops in the capital also contributes to exposure of people in their vicinity even if it is difficult to assess [13]. Communities are aware of their exposure to Hg, its link to gold mining, and the potential for neurological toxicity. However, the majority of individuals remain poorly informed about the precise causes, symptoms, and possible remedies [12]. 

Suppression of public health research on the impacts of mining and Hg contamination from gold mining by government and non-government organizations is obscuring the public health risks and leading to insufficient and misguided regulation. [Americaanse Wetenschapper Gevlucht uit Suriname: Na bedreiging ATM: Americaanse wetenschapper gevlucht uit Suriname (Ameriican Scientist Escaped from Suriname after threat from ATM) De West Newspaper, Paramaribo, Suriname, 19 July 2005]. Foreign researchers and in-country collaborators were warned of “dire consequences” if they communicate the effects on public and environmental health from Hg contamination from gold mining. The defenders of scientific censorship claim that the government has the right to set policy and deliver its own message in its own words. Under this system, research is highly institutionalized through disciplines and fields of knowledge. Research is also an integral part of political structures: funding agencies, universities, development programs, and policies. Research is regarded as being the domain of experts who have advanced educational qualifications and have access to highly specialized language, skills and resources [14].

While it is reasonable to require that scientists defend data and clarify statements, it is essential that research conducted at the intersection of race and economic class avoid the biases caused by Western systems for organizing, classifying, and storing new information, and for theorizing the meanings of such discoveries [14]. Indigenous communities are now calling for an end to “the ambulance at the bottom of the cliff approach to health” and are calling for support for projects using culturally appropriate, community-directed prevention and intervention strategies [2, 14]. This form of research is a pragmatic integration of both scientific and traditional knowledge systems. Western science focuses on hypothesis testing by data collection and statistical analysis. Indigenous traditional knowledge is based on cumulative experience, close observation, and oral knowledge communicated by elders and handed down over generations. This study worked with Wayana community leadership to create a collaborative environmental health research project. 

Wayana village leaders and participating villagers from Puleowime (Apetina) and Kawemhakan (Anapayke) in Suriname SE Lawa region (Figure 1) consistently prioritized three concerns facing their communities: (1) encroachment of their traditional homeland by miners who are heavily armed and prevent villagers from hunting, fishing, and providing for their families; (2) the effects of water and food chain contaminants from mining, especially nervous system damage from mercury (Hg) on their health; (3) clean water for drinking, cooking, and bathing is now scarce due to contamination from mining. (Testimony collected by authors during visit to Apetina 2008).

This study focused on item 2 and had one objective: Employ Participatory Action Research methods [15], participatory research methods [16], and the methods described by Smith [14] to conduct community-directed research so that two indigenous communities, Puleowime (Apetina) and Kawemhakan (Anapayke), with assistance from two charitable nonprofit organizations, could determine for themselves whether they were at risk from exposure to Hg contamination. 

## 2. Methods and Materials

### 2.1. Community-Led Research

In 2008, community members in Puleowime (Apetina) pointed to the frequency with which they have been overstudied and note that research conducted by the World Wildlife Fund in 2004 was done with little concern for community needs. [Personal communication from Leon Eric Wijngaarde, Director of Stichting Wadeken Wasjibon Maria and Sita Tempico, board member from Kawemhakan (Anapayke), 2008. (Stichting Wadeken Wasjibon Maria is a self-organized nonprofit foundation comprised of native Amerindian representatives from various communities in Suriname)]. In 2008, Stichting Wadeken Wasjibon Maria (SWWM) acquired the data from the 2004 study then met with village leaders to discuss community rights in research, data ownership and to interpret study results in terms of the health impacts of contaminant exposure. SWWM, a public health and indigenous advocacy non-governmental organization in Suriname, was then invited to meet with representatives from Kawemhakan (Anapayke) to discuss community needs. Leaders from both communities concluded that they wanted to determine for themselves whether they were at risk from exposure to Hg contamination and assess potential health impacts from Hg exposure, especially in children. Because community leaders had previously been criticized for releasing data to the press, they determined they wanted findings to be published in an “international peer-reviewed journal” that would be acknowledged as legitimate by domestic and foreign government health care agencies. Stichting Wadeken Wasjibon Maria (SWWM) requested the assistance of the Suriname Indigenous Health Fund (SIHF), a nonprofit non-governmental organization based in the United States, to provide technical expertise throughout the research process. A toxicologist, a sociologist, and public health physician represented SIHF on the research team.

The approach used was a combination of Participatory Action Research methods [15], participatory research methods [16], and the methods described by Smith [14]. These approaches drew on a Freirean approach that is a collaborative and collegiate process [17]. The elements of the community-led research process are as follows: (1) outside science experts were issued an invitation by community members to participate in a codirected study; (2) community members codeveloped a research plan according to appropriate scientific procedures and traditional cultural norms; (3) data was collected by trained community members; (4) data was owned and interpreted by community members; (5) the final determination for the disposition of research results was determined by community members according to traditional decision-making processes. 

Stichting Wadeken Wasjibon Maria, with support from the Suriname Indigenous Health Fund, held group discussions in Puleowime (Apetina) and Kawemhakan (Anapayke) to define roles and responsibilities and identify issues critical to establishing an effective, cooperative partnership between the scientists, health professionals, and the community. All meetings were held in the Wayana language.

### 2.2. Analysis of Hg Levels in Human Hair

Leaders from Puleowime (Apetina) and Kawemhakan (Anapayke) chose to collect hair samples for analysis (as opposed to blood or urine) because it is the least invasive sample to collect and the best indicator of dietary exposure to Hg from fish. Community members received training from SWWM educators and collected hair samples for analysis using methods designed to maximize sample quality and consistency and minimize cross-contamination, which emphasized the use of powderless surgical gloves and new, sterile, stainless steel scissors for each sample collected. Community members, with the assistance of representatives from the SWWM, collected and submitted hair samples for Hg analysis from 158 people in Puleowime (Apetina) and 106 people in Kawemhakan (Anapayke). Ages ranged from less than one-year to over 80 years old in both communities. In Puleowime, participants was more female (92) than male (67). In Kawemhakan, the number of males and females were almost the same (54 and 52, resp.). All hair samples were collected from the lower occipital region. When long hair strands (>3 cm) were collected, the hair tips were discarded and only the proximal 1 cm were used.

Each hair sample, of approximately 20 mg, was placed in a labeled envelope. The hair samples were analyzed in triplicate for total Hg by a SIHF technician educated in analytical chemistry and trained in the operation of the Lumex Hg analyzer. Hg analysis was by the cold-vapor technique using the Portable Zeeman Lumex (RA915^+^/RP-91C) mercury analyzer. The instrument detection level was 0.2 ng/g. All concentrations were expressed in parts per million (equal to *μ*g/g Hg). Measurement of Hg levels in hair using the Lumex RA915^+^/RP-91C portable analyzer had been previously confirmed by laboratory analysis using a modified National Institute for Occupational Safety and Health (NIOSH) 6009 method. In this study, the Lumex was operated in software “On Stream” mode using the procedure in the manufacturer's operation manual. NIST traceable standards 2709 for Hg at 1400 ng/g and 1633d for Hg at 141 ng/g were used to standardize the analyzer before and after each ten samples analyzed.

SWWM and SIHF consulted with the communities throughout the process. Following the analysis of all hair samples, meetings were held to discuss the results and answer any questions the community had regarding the data. Afterwards, a community meeting was held to reflect on the process, outcome, and future needs. A physician was in attendance to answer questions from the community. The attending physician performed limited examinations on persons who requested a consultation because they thought they had been exposed to potentially hazardous levels of mercury. The purpose of this examination was to address their concerns, alleviate anxiety, determine whether their concerns had merit, and provide a baseline for future health monitoring. The examination by the physician included a discussion regarding the patients medical history, with emphasis on the nervous system (target organ for chronic exposure), the kidneys (target organ for acute and chronic exposure), the oral cavity (target organ for chronic exposure), the lungs (target organ for acute exposure), the eyes (affected by chronic exposure), and the skin (since mercury is a known skin sensitizer). Early signs and symptoms of mercury intoxication were elicited by employing finger-to-nose testing, rapid alternating hand movements, and diminished two-point discrimination tests. 

The guidelines used to interpret the significance of an individual's results were the following.

1. If a person's laboratory results were less than 1 *μ*g/g, they were told that the Hg level in their hair was below the recommended upper limit. The United States Environmental Protection Agency (EPA) [18] recommends that safe levels of Hg found in hair are below 1 *μ*g/g.
2. If a person's laboratory results were between 1 *μ*g/g and 11 *μ*g/g, they were advised that their Hg level was above the recommended limit. They were also told that they could be at elevated risk if they were pregnant, planning to become pregnant, or nursing a baby. They were advised to seek the advice of a medical professional if they had any health concerns.
3. If a person's laboratory results were greater than 11 *μ*g/g, they were told that their Hg level was above the benchmark dose set by the EPA. They were also told that they could be at elevated risk if they were pregnant, planning to become pregnant, or nursing a baby. They were advised to seek the advice of a medical professional.

Regardless of the other languages understood by the people in (Apetina) and (Anapayke), that is, Dutch and Sranan Tongo, they assert that they are only able to fully understand public health information when the material is translated into their native Wayana language. As a consequence of this observation, the communities are currently working with Stichting Wadeken Wasjibon Maria, with support from the Suriname Indigenous Health Fund, to develop more detailed education programs that explain the importance of the guidelines for pregnant women and children in the Wayana language.

### 2.3. Data Analysis

In this study, hair mercury results were summarized using simple descriptive statistics including arithmetic mean, median, standard deviation, and range. The mean hair concentrations were evaluated by population, age and gender using the two-tailed *t-*test assuming equal variances (*P* < 0.05). Geometric mean and standard deviation were used for the analysis of exponential data related to risk. 

In this report, the concept of a benchmark dose (BMD) is used to calculate risk. The BMD is the estimated dose corresponding to a specified incremental risk over and above background. Individual risk, defined here as the probability of having a 5% chance of exhibiting an adverse neurological effect, was based on the most conservative of the three dose response functions (DRFs) reported by Sullivan et al. [19] in which risk is correlated to the biomarker of Hg concentration in hair as a function of the amount of Hg consumed through fish. According to Sullivan, the probability of having a 5% chance of exhibiting an adverse neurological effect was estimated to be 0 for hair at 0–3 ppm Hg, 1 × 10^−4^ for hair at 4 ppm, 1 × 10^−3^ for hair at 5-6 ppm, 2 × 10^−3^ for hair at 7 ppm, 3 × 10^−3^ for hair at 8 ppm, 5 × 10^−3^ for hair at 9 ppm, 1 × 10^−2^ for hair at 10 ppm, 1 × 10^−1^ for hair at 11 ppm, 4 × 10^−1^ for hair at 12 ppm, 6 × 10^−1^ for hair at 13 ppm, and 9 × 10^−1^ for hair over 13 ppm. Population risk was obtained through the summation of risk for all individuals [19, 20]. Risk assessors use the term “population risk” to mean the number of people in the community that are affected. By contrast, “individual risk” is the incremental probability that the hazard will impose an effect on some particular person [21].

### 2.4. Hazard Quotient

Exposure can be expressed as a noncarcinogenic risk expressed in terms of the hazard quotient (HQ) [19]. The ratio of the exposure value (dietary intake, *μ*g/kg/day) to the risk value (RfD) provides an estimate of risk. In 2004 the Joint FAO/WHO Expert Committee on Food Additives (JECFA) established a tolerable intake of 1.6 *μ*g/kg bodyweight per week (0.23 *μ*g/kg bw/d) for mercury in order to protect the developing fetus from neurotoxic effects [22]. 

The mean conversion factor from consumption (*μ*g/kg/d) to hair Hg (*μ*g/g) was assumed to be 10 [23–25]. If the quotient is one or more then an adverse effect is likely to occur. Reference levels are indicators of the potential for adverse effects when consistently exceeded. When the hazard quotient is less than one, the mercury exposure could be regarded as unlikely to lead to adverse health effects. 

As exposures increase above the reference level, either by magnitude or by time, the likelihood of adverse effects also increases. Generally, if the hazard quotient is greater than 1, more evaluation is warranted to determine the degree and frequency of exposures above the reference level. Although the quotient method is commonly employed, it is the least probabilistic of the methods used and is highly dependent on professional judgment.

### 2.5. Professional Judgment and Indigenous Wayana Judgment

Risk assessments are based on scientific data that are frequently difficult to interpret and complex, conflicting or ambiguous, and incomplete. Analysis of this data for risk assessment purposes depends, therefore, on professional judgment based on scientific expertise. The analysis of this data for risk assessment purposes also depended on indigenous Wayana judgment because the communities performing the risk assessment are embedded in a social, political, and economic context that shapes the behaviors of the stakeholders that are involved, and determines the community access to resources that are necessary to maintain health. Together, outside advisors and Wayana leaders designed the risk assessment plan, evaluated methods to be used, and interpreted the significance of exposure data and the observed effects. 

Participatory research differs from conventional research in the alignment of power within the research process [17]. Methodologically, outside experts became learners, facilitators, and catalysts in a process that gathered momentum as the community came together to analyze and discuss the research process and the results it yielded. The process was characterized by a cycle of dialogue, reflection, and action. This method relied less on the methodological framework than it did on the relationship between the researchers and the community. The program was managed in a manner that ensured that all partners' interests and aspirations were considered, and activities were implemented only with agreement from all partners involved. The relationship followed here was described by Biggs [26] who described this mode of community participation as *collegiate *where *researchers and local people work together as colleagues with different skills to offer, in a process of mutual learning where local people have control over the process.*

### 2.6. Human Subjects Review

The SIHF research team consulted with the human subjects review staff at the University of Washington who approved the project plan on 18 June 2007 and again on 30 December 2010 to review plans to publish this paper. The Institutional Review Board staff found that the research design did not require full IRB review since the traditional roles of researcher and research subject do not apply. Since research subjects were co-investigators leading the research process while the western research team acted as consulting technicians, informed consent was deemed unnecessary. 

## 3. Results

The estimated risk of adverse neurological effects at measured levels of Hg in hair are given in Table 1. In both communities, 58% of the people who submitted hair samples had Hg levels above the World Health Organization [27, 28] safety limit (10 *μ*g/g). The mean hair Hg concentration in Puleowime (GM = 14) was significantly higher (*P* < 0.05, df 262) than the hair Hg levels in Kawemhakan (GM = 8). Although mean hair concentrations varied from 11 ± 6 to 20 ± 6 *μ*g/g, the differences were not significant at the 95% confidence level (Table 2). The population risk for Puleowime was 93 and for Kawemhakan was 18, which reflects the number of people in each community expected to be affected by exposure to excess levels of mercury.

Facilitators from SWWM also noted information related to fish consumption patterns and self-reported symptoms. In Puleowime, all participants reported eating fish at least 3 times per day every day whereas, in Kawemhakan, more than 25% of the participants reported consuming fish less frequently. Residents in Puleowime (Apetina) and Kawemhakan (Anapayke) reported that the two most common varieties of fish eaten in their communities were *anumara* (*Hoplias spp*.) and *tucunare (Cichla sp.).*

Among participants in Puleowime, 12% reported feeling numbness in arms, fingers, or toes. In Kawemhakan, that number was higher at 36%. Three women with hair mercury levels between 25 and 30 ug Hg/g requested a health assessment. All complained of headaches and pain and tingling in their hands and feet. The attending physician, D. J. Roesel, Clinical Assistant Professor, General Internal Medicine and Adjunct Clinical Assistant Professor, Global Health at the University of Washington, noted that three women who presented themselves to him with concerns that they were affected by exposure to mercury exhibited abnormal neurological testing, with poor performance on finger-to-nose testing, rapid alternating hand movements, and diminished two-point discrimination. In both Puleowime and Kawemhakan, over one-third of the participants complained of either headaches or strong feelings of sadness or depression at least once weekly. 

## 4. Discussion

This study was successful in reaching its primary objective. Two Wayana communities in southeast Suriname successfully completed a project in which they measured for themselves their risk of exposure to mercury (Hg) contamination using a model of community-led research. 

Mercury exposure in the Wayana villages of Puleowime (Apetina) and Kawemhakan (Anapayke) appears to mirror the problems documented elsewhere in the region. In two Wayana villages in French Guyana, 58% and 57% of the people had Hg levels above the World Health Organization (WHO) safety limit (10 *μ*g/g), respectively [27, 28]. Frery et al. [11] measured hair Hg concentrations in 235 samples from people in four villages along the upper Maroni River in French Guiana where the average concentration was 11 ± 4 *μ*g/g (mean ± SD). This value corresponds to the exposure levels in Kawemhakan where the average hair Hg concentration was 8 ± 4 *μ*g/g. In Puleowime, however, the average hair Hg concentration was significantly higher at 14 ± 6 *μ*g/g (*P* < 0.05). 

Frery et al. [11] conducted a detailed familial dietary study associated with Hg measurements in fish and some game. The Frery study was conducted over 7 days in two different seasons in the four most populated Wayana villages on the upper part of the Maroni River. The results confirm mercury exposure of the Wayana population related to a diet rich in fish, which are highly contaminated for certain species (up to 1.62 mg/kg fresh weight or 8.1 mg/kg dry weight in skeletal muscle). Frery showed that Hg concentrations in fish muscle were closely linked to the feeding regime and position of fish in the food webs. Overall, 14.5% of the fish collected exceeded the 0.5 mg/kg (fresh weight) safety limit. Four carnivorous species accounted for no less than 72% of the metal ingested by the Wayana families, although these represented only 28% of the consumed fish biomass. The species were *Pseudoplatystoma fasciatum* (27% of the Hg dietary intake), *Hoplias aimara* (27%), *Ageneiosus brevifilis* (11%), and *Serrasalmus rhombeus* (6.5%). *Cynodon meionactis*, which along with *P. fasciatum* has the highest Hg concentrations, was hardly eaten at all (especially by children) because these fish contain a large number of bones. The two species that are consumed in the greatest amounts, *Myleus rhomboidalis/tometes *(12.7%) and *Doras micropeus* (11.2%), are not contaminated to a very high degree (100 and 1,160 (micro) g/g, dw, resp.). The Frery study revealed excessive exposure to mercury in the Wayana population was related to the consumption of contaminated fish.

While several studies have shown that Hg levels in hair are higher in residents of areas contaminated by mercury than in residents of uncontaminated regions, others show wide variations depending on the relative importance of fish in the diet [5]. In Puleowime, the community fills more of its dietary needs by fishing than Kawemhakan where the residents are more acculturated and have a more diversified diet. In both cases, subsistence fishers consume large amounts of fish and represent high exposure cases that form the tail of the distribution of the general population. The Wayana live in isolated villages on the Tapanahoni River (Apetina) and the Lawa River (Anapayke) and are considered excellent examples of members of a “fishing civilization.” Recent investigations by Frery et al. [11] show that most subjects take more than 14 fish meals per week. The actual exposure, therefore, among these populations will be highly variable, location specific, and they will depend on local fish Hg levels and individual fish consumption patterns. 

 In both Puleowime and Kawemhakan the risk of having an adverse effect is quite large (population risk 93 and 18 resp.). In general, a lifetime risk of one-in-ten-thousand and in some instances one-in-one million has become a common place standard in public health discourse and policy. The risks for subsistence fishers in Puleowime and Kawemhakan are orders of magnitude greater than the risk that is acceptable in mainstream society. 

Another way to consider risk is by comparing the estimated oral exposure dose (*μ*g/kg/d) to an oral reference level to calculate the hazard quotient (HQ) [23–25]. For Puleowime, the dietary exposure rate was 1.4 *μ*g/kg/d and for Kawemhakan, 0.9 *μ*g/kg/d. The FAO/WHO Expert Committee on Food Additives (JECFA) reference level for dietary exposure to Hg is 2.3 *μ*g/kg/d [22]. Both yield hazard quotients greater than 1 as did the individual exposure doses based on measured hair Hg concentrations (Table 1). Of these, approximately 15% were children under the age of 5 and 34% were women of childbearing age. These people are especially susceptible to mercury exposure because of the sensitivity of the developing nervous system in the fetus and in children. 

From the point of view of the physician who was in attendance, there are many potentially confounding factors that are affecting community health. As a global and public health professional, the research team physician struggled with the idea that, although there are compelling instances of high levels of mercury exposure, there was no “smoking gun” in terms of clearly caused, well-documented health impacts from mercury exposure. The health impacts observed can also be related to social decay, loss of hunting grounds and land rights, and the threat of extinction of these indigenous communities and their way of life. Consequently, when considering the impacts of contamination from mining it is important to consider the broadest definition of “health” possible [23, 24]. This approach argues in favor of long-term community partnerships over short-term public health campaigns that are iterative in nature and open to the possibility of collaboration with other disciplines that can address a broader range of social and legal considerations.

## 5. Wayana Community Review of Report

Research data on the exposure of Wayana people to mercury has been reported since before 1994 [7]. Since then, research and outreach has been conducted by government and non-government organizations including a study conducted in 2004 by the World Wildlife Fund. As late as 2007, the results of this research had not been published nor returned to the Wayana communities. 

There were two ways in which the community-led approach to research was different from research performed previously among the Wayana. The first was that the Wayana communities were equal partners with Western-trained experts. The community-led approach involved a collaboration of “formally trained research” partners from the fields of environmental toxicology, sociology, and public health as well as indigenous advocates and community leaders. As the experts in their environmental and cultural context, community leaders were able to identify the research question, select a feasible and culturally appropriate research strategy, interpret data findings within their own social structure, and identify next steps within traditional decision making mechanisms. Second, instead of creating knowledge for knowledge's sake or instead of performing research for the advancement of the field of risk assessment or toxicology, the community-led approach was an iterative process that incorporated research, reflection, and action in a cyclical process for the benefit of the communities at risk. 

According to Aptuk Noewahé, the Wayana Granman (leader) from Puleowime (Apetina), past research was conducted by scientists who, “came often, said big things, made promises, then left.” He said that if scientists want to help, then they should “include the community, listen and help. Otherwise, they should just go away”. As Cornwall and Jewkes [17] argue “participatory research consists less of modes of research which merely involve participation in data collection than of those which address issues of the setting of agendas, ownership of results, power and control.” 

After the risk assessment analyses were complete, the results were discussed with the community. The communities asked Suriname Indigenous Health Fund and Stichting Wasjibon Wadeken Maria to help write this report communicating results of the community-directed risk assessment. The report was drafted in English, translated into their native language (Wayana), and reviewed. Regardless of the other languages understood by the people in Puleowime and Kawemhakan they were only able to fully understand the details of this complex problem when the report was translated into their native language for their review, comment, and approval. As a consequence of this process, the communities now want to repeat the education programs presented previously by government and NGOs, this time in their Wayana language.

Noewahé added that, “We support the article and the research because we led the process as partners in the project, and we are involved. Usually people do not discuss their work with us, not even the results of their work. This article is important because our problem needs to be known by others. Also, we look forward to continuing this project and we hope that together we can work towards a sustainable solution to our problem together with the government and other health organizations.”

Community elders agreed that, “this was a good project, because it means our children could have a good future.” However, there were a lot of other comments that were detailed in nature and reflected the complexity of the problem. The first was language. As a consequence of engaging the community in the conduct of this risk assessment project and discussing the data and drafts of this manuscript in the community's native Wayana language, one participant noted that, “Now we understand the problem and want to repeat the education programs presented previously by the government and NGOs, this time in our Wayana language.” Residents challenged suggestions made by Westerners that communities impacted by mercury (Hg) contamination must eat small, young fish from the lowest possible trophic level; residents noted they do not often have a choice. “We eat what the river gives us. We tried eating only the fish we were told to eat but could not catch enough and after 3 or 4 months we had to quit trying.” Instead, communities want to focus on integration, education, land rights, human rights, and the root causes leading to a wider range of problems that includes risk from exposure to mercury.

Community leaders identified three major priorities for further investigation. First, the Wayana communities of Puleowime (Apetina) and Kawemhakan (Anapayke) prioritized the assessment of potential health impacts from Hg exposure. Health assessments must be conducted for all community residents, beginning with the most susceptible subpopulations. Second, further risk assessments to evaluate the degree and frequency of exposures above the reference level must be conducted since residents of these communities consume larger amounts of fish than mainstream communities. Third, the major concerns facing the communities voiced throughout the research process that were not addressed in this study must be addressed. These include the encroachment of their traditional homeland by miners who are heavily armed and prevent villagers from hunting, fishing, and providing for their families; and the scarcity of clean water for drinking, cooking, and bathing due to contamination from mining.

On its own, a risk assessment study can serve as an example of reductionism and illustrates the approach that forms the basis for modern science. In many cases, a good understanding of the components of a system will lead to a good understanding of the system as a whole. However, within the context of an indigenous society where the relationship between the environment and human population is complex and multilayered, emergent properties of the system are impossible to predict from knowledge of discrete parts, and a holistic rather than a reductionist approach is necessary. The community-led research process provides a method for bridging the disciplines within scientific paradigms with traditional indigenous paradigms. The Wayana have a cosmology, which is distinct from the Western Reductionist approach to science. It integrates society, nature, and health and has more in common with Western Complexity theory, systems thinking and a holistic paradigm. By allowing community members to take a lead role in the research process, community-led research facilitates the exchange of information and shared decision-making that provides access to public health information for both at-risk communities and researchers. 

## 6. Conclusion

In this study the Wayana communities of Puleowime (Apetina) and Kawemhakan (Anapayke) in Suriname identified for themselves that they were at a high lifetime risk of adverse effects from exposure to mercury. Exposure was higher in Puleowime (Apetina) compared to Kawemhakan (Anapayke) where differences in the relative importance of fish in the diet may be responsible. Risk estimates suggest that all participants in the study exceeded the one-in-ten-thousand policy common in mainstream society. 

The community-led research process allowed community leaders to identify the research question most meaningful to them, select a feasible and culturally appropriate research strategy with the assistance of trained experts, collect data from participants within their community themselves, interpret data findings within their own social structure, and identify next steps within traditional decision making mechanisms. 

The community-directed approach increases the visibility of researcher and the transparency of their intentions, which are significantly greater than in conventional research. In practice, community-directed research was not a simpler alternative to a conventional research project and working in collaboration with local people is far from easy. Contrary to expectations, control over research does not devolve completely onto the community, nor do communities want to assume complete control. 

## Figures and Tables

**Table 1:** Total hair mercury (Hg) concentrations (*μ*g/g) and risk by population (community) and subgroup (age and gender) among residents of Puleowime (Apetina) and Kawemhakan (Anapayke).

Community/age group	Average age	No.	Geometric mean Hair hg (*μ*g/g) ± SD	Median hair Hg (*μ*g/g)	Hazard quotient (HQ)	Range hair Hg (*μ*g/g)	Geometric mean of individual risk
Puleowime (Apetina)	24	158	14 ± 6	14	6.0	3–34	0.23
≤5 years	3	23	17 ± 7	20	7.0	5–28	0.25
Male	2	9	20 ± 6	23	9.0	7–28	0.45
Female	16	14	15 ± 7	16	6.0	5–26	0.18
6–14 years	9	53	14 ± 5	14	6.0	6–34	0.26
Male	9	27	16 ± 6	15	7.0	8–34	0.42
Female	10	26	13 ± 5	14	6.0	6–25	0.15
15–25 years	20	19	14 ± 7	14	6.0	6–32	0.16
Male	22	5	13 ± 5	14	6.0	6–18	0.20
Female	19	14	14 ± 8	13	6.0	8–32	0.15
26–45 years	35	35	15 ± 7	14	6.0	6–33	0.22
Male	35	13	15 ± 8	13	6.0	7–33	0.27
Female	34	22	14 ± 7	15	6.0	6–30	0.20
>45 years	59	28	13 ± 6	13	6.0	3–29	0.22
Male	58	13	11 ± 6	13	5.0	3–29	0.22
Female	62	15	14 ± 5	14	6.0	7–27	0.21
Population risk							93

Kawemhakan (Anapayke)	28	106	9 ± 4	9	4.0	2–19	0.01
≤5 years	3	17	9 ± 4	10	4.0	2–18	0.02
Male	3	9	8 ± 3	10	4.0	2–14	0.01
Female	3	8	10 ± 4	9	4.0	6–18	0.02
6–14 years	9	27	5 ± 2	6	2.0	2–9	0.00
Male	9	13	6 ± 2	5	3.0	3–9	0.00
Female	9	14	5 ± 2	6	2.0	2–8	0.00
15–25 years	22	8	8 ± 3	8	4.0	4–16	0.01
Male	22	6	8 ± 4	9	4.0	4–16	0.00
Female	22	2	9 ± 1	9	4.0	9-10	0.01
26–45 years	35	28	8 ± 4	8	4.0	4–18	0.01
Male	36	17	9 ± 4	9	4.0	4–18	0.03
Female	34	11	7 ± 2	7	3.0	4–11	0.00
>45 years	59	26	10 ± 4	10	4.0	5–19	0.02
Male	61	9	11 ± 4	13	5.0	5–18	0.07
Female	59	17	9 ± 4	8	4.0	6–19	0.01
Population risk							18

**Table 2:** Summary of *t*-test statistics supporting analysis of hair mercury concentrations in residents from Puleowime (Apetina) and Kawemhakan (Anapayke).

	Data category	Mean	*df*	*P*
Community	Apetina	16		
	Anapayke	9	262	7.80*E*−20
Gender	Apetina-Female	15		
	Apetina-Male	16	158	0.19
	Anapayke-Female	8		
	Anapayke-Male	9	105	0.21
Age	Apetina-≤5 years	18		
	Apetina->5 years	16	56	0.27
	Anapayke-≤5 years	10		
	Anapayke->5 years	9	105	0.26
